# The Relationship Between Cognitive Behavioral Therapy and Post-Traumatic Growth: A Systematic Review

**DOI:** 10.3390/healthcare14131857

**Published:** 2026-06-25

**Authors:** Dimitrios Kasimis, Paschalia Mitskidou, Athanasios Tselebis, Ioannis Ilias, Argyro Pachi

**Affiliations:** 1 Psychiatric Department, Sotiria General Hospital of Chest Diseases, GR-11527 Athens, Greece; kasimisdimitrios@yahoo.com (D.K.); irapah67@gmail.com (A.P.); 2 Behaviour Therapy Unit (BTU), Hellenic Center of Mental Health and Research, GR-11526 Athens, Greece; dr.mitskidou@outlook.com; 3 Department of Endocrinology, Hippokration Hospital, GR-11527 Athens, Greece; iiliasmd@yahoo.com

**Keywords:** cognitive behavioral therapy, post-traumatic growth, post-traumatic stress disorder, systematic review, CBT-based interventions, PRISMA, trauma, psychological well-being

## Abstract

**Background:** Post-traumatic growth (PTG) refers to positive psychological changes resulting from the struggle with highly challenging or traumatic life events. Psychosocial interventions have demonstrated efficacy in promoting psychological well-being in the aftermath of traumatic experiences. Cognitive Behavioral Therapy (CBT) is among the most extensively studied such interventions, aligning with the PTG model’s prerequisites for growth. **Objective:** The aim of this systematic review was to assess the efficacy of CBT and CBT-based interventions in promoting PTG. **Methods:** A systematic review was conducted in accordance with the Preferred Reporting Items for Systematic Reviews and Meta-Analyses (PRISMA) guidelines, searching PubMed, Scopus, and Google Scholar databases from inception to December 2024. Eligibility criteria included: (a) the inclusion of a CBT or CBT-based intervention, (b) measurement of PTG using the Post-Traumatic Growth Inventory (PTGI), (c) study participants having experienced traumatic life events, and (d) articles written in English. Risk of bias was assessed independently by two reviewers. Due to the heterogeneity of included studies, a qualitative narrative synthesis approach was adopted. Risk of bias was assessed using the RoB-2 tool for RCTs, ROBINS-1 for quasi-experimental studies and Newcastle–Ottawa scale for cohort studies. Certainty of evidence, assessed using the GRADE framework, is considered low. **Results:** A total of 19 studies were included (13 randomized controlled trials, 3 quasi-experimental, and 3 longitudinal studies). While traditional CBT produced mixed results in fostering PTG, CBT-based therapeutic protocols—particularly those explicitly designed to target PTG or incorporating structured cognitive–emotional techniques—demonstrated more consistent benefits. Limitations of the included studies include measurement of PTG as a secondary outcome, small sample sizes, and the presence of confounding variables. **Conclusions:** Further high-quality, multicenter randomized controlled trials with standardized protocols are needed to clarify the role of CBT in promoting growth after trauma.

## 1. Introduction

The field of traumatology has traditionally focused on the detrimental psychological and physical consequences of traumatic experiences and their treatment. Nevertheless, positive transformation following exposure to traumatic circumstances has been observed for decades, and has long been a subject of clinical and scientific interest. Several constructs have been proposed to capture this phenomenon: perceived benefits [1], defined as self-reported positive life changes following adversity; positive changes [2]; stress-related growth [3], describing positive outcomes resulting from severe life events; and thriving [4], referring to positive psychological adaptations arising from coping processes following stressful events. However, the most widely adopted model describing positive psychological consequences after trauma is that of post-traumatic growth (PTG).

### 1.1. Conceptual Background of the Post-Traumatic Growth Model

Tedeschi and Calhoun first described the concept of PTG in 1995, defined as positive psychological changes experienced due to struggle with trauma or highly challenging situations [5]. Growth pertains to a transformational process that exceeds the individual’s pre-existing psycho-social functionality, on interpersonal, intrapersonal and spiritual/existential domains [5,6,7,8]. This core characteristic differentiates the concept of PTG from similar trauma-related psychological outcomes such as resilience. Growth stems from the ability to successfully cope with trauma, an unforeseen life-altering event that challenges pre-existing assumptions and core beliefs, in the aftermath of cognitive, emotional, and social struggle [6,7]. Consequently, successful coping requires a cognitive restructuring of these beliefs and a deliberate reframing of the traumatic narrative [8]. Critical to this narrative reconstruction is the shift from intrusive rumination—which is characteristically distressing and tied to post-traumatic stress symptoms—to deliberate, constructive rumination [5,6,7,8]. While these two forms of cognitive processing are not mutually exclusive, the transition toward deliberate reflection, acceptance, and reframing is mediated by adaptive coping mechanisms and social support [8]. Crucially, because the PTG framework posits emotional distress as a prerequisite for growth, the model underscores that distress and positive psychological change are not opposite ends of a spectrum; rather, they co-exist within the individual in the aftermath of trauma [8,9].

As researchers sought to operationalize PTG as a quantitative outcome, the Post-Traumatic Growth Inventory (PTGI) was developed [6]. The PTGI is a 21-item scale measuring growth across five dimensions: Personal Strength, Relating to Others, New Possibilities, Appreciation of Life, and Spiritual and Existential Change [6,7,8]. Even though PTGI remains the most widely adopted method of PTG measurement across the literature, researchers have proposed alternatives to the five-factor structure of the original scale [10], while others have questioned its validity in reporting actual positive change, given its self-report nature.

It is important to acknowledge that PTG is not a simple or uncontroversial construct. Debate exists in the literature concerning what PTG actually reflects: genuine psychological transformation, perceived benefit, meaning-making as a coping strategy, or a self-protective reinterpretation of difficult experiences [8,11]. A person may experience a reduction in distress without achieving the kind of cognitive and existential reconfiguration described in the PTG literature; conversely, growth and distress can co-occur [11]. Nevertheless, recent studies clarify the distinction between PTG and perceived benefits, with growth encompassing positive changes in cognitive, emotional and social functioning, beyond merely a subjective sense of well-being [9].

A clinically relevant question that has attracted considerable research attention is whether PTG can be facilitated by psychosocial interventions. Tedeschi et al. note that positive therapeutic outcomes in clinical psychology often correspond to positive psychological changes consistent with the PTG model [8], arguing that symptom reduction is not identical to nor necessary for growth. Moreover, Roepke’s 2014 meta-analysis [12] concluded that diverse intervention approaches, even when growth is not the explicit treatment target, can foster PTG [12].

### 1.2. Theoretical Basis of Cognitive Behavioral Therapy for PTG

Extensive meta-analytic evidence positions trauma-focused Cognitive Behavioral Therapy (TF-CBT) as a gold-standard modality for trauma-related distress, earning it top-tier recommendations across multiple international clinical guidelines [13]. Standard TF-CBT protocols rely on specific, manualized components, including psychoeducation, emotional regulation skills, exposure techniques (both imaginal and in vivo to target avoidance), and cognitive restructuring to address distorted, trauma-related appraisals [13].

While traditionally evaluated by its capacity to reduce clinical deficits, the theoretical rationale for expecting CBT to actively promote PTG rests on its structural alignment with shattered-assumption frameworks. Tedeschi et al. [8] assert that PTG emerges from a transition away from intrusive, unconstructive thoughts toward deliberate rumination—the purposeful, reflective processing of a traumatic experience. This cognitive reassessment and meaning reconstruction is central to CBT; cognitive restructuring directly facilitates the deep schematic changes and core-belief modifications that underlie growth [14,15].

Beyond restructuring, specific CBT mechanisms correspond directly to established predictors of PTG. First, exposure protocols promote approach-oriented coping rather than avoidance, providing a direct behavioral pathway to growth [14,16,17]. Second, by equipping individuals with active, problem-focused coping strategies, CBT systematically enhances self-efficacy [18,19,20]. Finally, the clinical reduction in acute trauma symptoms serves as a vital mediator for growth [21]. By down-regulating overwhelming autonomic and emotional arousal, CBT brings post-traumatic distress down to a manageable, moderate threshold—the exact cognitive bandwidth required for patients to engage in productive meaning-making and value exploration [16].

Given CBT’s proven efficacy, mechanistic compatibility with PTG, and protocol versatility, this systematic review aims to assess recent evidence concerning the facilitation of PTG specifically through CBT and CBT-based interventions across diverse traumatic experiences, populations, and delivery formats. Ultimately, these findings may enhance clinical practice by clarifying the utility of growth as an explicit treatment outcome, optimizing the integration of PTG-focused components within standard CBT protocols, and establishing a rigorous empirical basis for future research directions.

## 2. Materials and Methods

This systematic review was conducted (Supplementary File S1) in accordance with the Preferred Reporting Items for Systematic Reviews and Meta-Analyses (PRISMA) guidelines [22] and has been prospectively registered in the PROSPERO International Prospective Register of Systematic Reviews (Registration ID: CRD1337609).

A comprehensive literature search was conducted in PubMed, Scopus, and Google Scholar from database inception to December 2024. The following search equations were used:

PubMed/Scopus: (“Cognitive Behavioral Therapy” OR “CBT” OR “Cognitive Behavioural Therapy” OR “Cognitive Behavioral Intervention” OR “Cognitive” AND “Behav*”) AND (“Post-Traumatic Growth” OR “PTG” OR “Positive Psychological Changes”).

Google Scholar: “CBT” AND “PTG”; “Cognitive Behavioral Interventions” AND “Positive Psychological Changes”; “Cognitive” AND “Behav*” AND “post-traumatic” AND “growth”.

**Figure 1:**
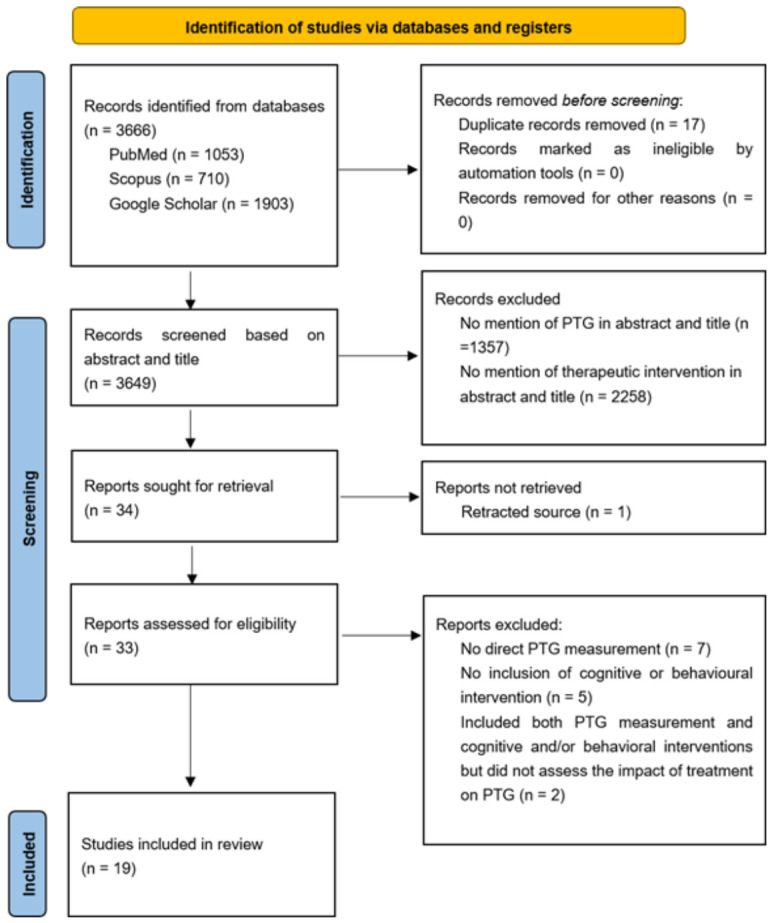
PRISMA flow diagram of systematic search results.

Boolean operators AND and OR were applied across all databases. No language restrictions other than English were applied at the database level, but studies not written in English were excluded at the screening stage. The initial search yielded 3666 articles. After de-duplication across databases, 3649 articles remained. Abstracts and titles were independently screened by two reviewers against the eligibility criteria, resulting in the exclusion of 3615 articles. Of the 34 remaining full-text articles, one study was retracted, hence 33 articles were retrieved and assessed for eligibility. After independent review and unanimous exclusion of 14 articles not relevant to the review topic, 19 studies were included in the final synthesis (Figure 1).

### 2.1. Eligibility Criteria

Studies were eligible if they: (a) included participants who had experienced a traumatic life event (as defined by the DSM or reported by study authors); (b) delivered a CBT or CBT-based intervention (a diversity of protocols that include any form of cognitive restructuring and/or behavioral techniques including trauma-focused CBT, internet-based CBT, group cognitive–emotional training and MBCT); (c) measured PTG using the PTGI as a primary or secondary outcome; and (d) were written in English. Studies were excluded if they: (a) did not assess PTG or a CBT-related intervention; (b) did not examine or infer a link between the two; or (c) were themselves systematic reviews, literature reviews, or meta-analyses.

### 2.2. Data Extraction

Data extraction was performed by two independent reviewers using a pre-specified Excel spreadsheet capturing: date of publication, authors, country, study design, participant characteristics, type of trauma, type of CBT intervention, comparator group(s), PTG measurement tool and timing, and main findings (including effect sizes or statistical significance of PTGI score differences). Disagreements were resolved by consensus.

### 2.3. Risk of Bias Assessment

Risk of bias for randomized controlled trials was assessed using the Cochrane Risk of Bias 2 (RoB 2) tool [23], evaluating domains of randomization, deviations from intended interventions, missing outcome data, measurement of the outcome, and selection of reported results. For quasi-experimental studies, the Risk of Bias in Non-Randomized Studies of Interventions (ROBINS-I) tool was applied [24], and for longitudinal observational studies, the Newcastle–Ottawa Scale (NOS) [25] was used (Supplementary File S2, Tables S1–S3). Two independent reviewers conducted assessments, with disagreements resolved by consensus. Overall, most RCTs presented low to some concerns regarding risk of bias; the primary concerns across studies were inadequate allocation concealment, lack of blinding, small sample sizes, and measurement of PTG as a secondary rather than primary outcome. Quasi-experimental studies were at higher risk of bias due to absence of randomization and limited control for confounders. Longitudinal studies lacked control groups, limiting causal attribution of change. Comprehensive tables reporting risk of bias assessment for each individual study are included as complementary material.

### 2.4. Certainty of Evidence

The certainty of the overall body of evidence was rated using the GRADE (Grading of Recommendations Assessment, Development and Evaluation) approach [26]. Starting from the assumption of high certainty for RCTs, certainty was downgraded due to: (a) serious risk of bias in several included studies (e.g., lack of blinding, allocation concealment concerns); (b) inconsistency of results across studies (heterogeneity of interventions and outcomes); (c) indirectness (PTG was a secondary outcome in many studies); and (d) imprecision (small sample sizes in most studies). Accordingly, the final overall certainty of evidence was rated as low, with traditional CBT studies yielding lower certainty and novel CBT-based, PTG-targeted protocols yielding moderate certainty. A comprehensive table reporting GRADE assessment by study design and intervention type created using the GRADEpro Guideline Development Tool Software, McMaster University and Evidence Prime, 2026 [27], is included as complementary material (Supplementary File S2, Table S4.).

### 2.5. Synthesis Approach

Due to marked heterogeneity among included studies—in terms of intervention type, population, trauma context, comparator, and outcome measurement timing—a meta-analysis was not feasible. A qualitative narrative synthesis was conducted, organized by intervention type (traditional CBT and CBT-related protocols; internet/telephone-based CBT; and studies in children, adolescents, and young adults), with attention to methodological quality, role of PTG as primary vs. secondary outcome, comparator type, and effect magnitude.

## 3. Results

A summary of all included studies is presented in Table 1, organized by intervention category.

### 3.1. PTG Facilitation by CBT in Adults

#### 3.1.1. Traditional CBT and CBT-Related Interventions

A single study reporting a significant positive relationship between CBT-based interventions and PTG as measured by the PTGI is that of Wagner et al. [28]. Researchers conducted an RCT examining the effects of Cognitive Behavioral Conjoint Therapy for PTSD (CBCT for PTSD) in 40 couples, each including one participant with PTSD following various traumatic experiences [28]. The treatment group demonstrated significantly increased PTGI scores over time compared to the waitlist control group, with a moderate effect size [28]. Notably, pre-treatment PTSD symptom severity was not associated with PTG outcomes [28].

Five additional studies incorporated CBT techniques targeting specific trauma-related disorders, particularly PTSD and prolonged grief, and found significant PTGI improvements [21,29,30,31,32]. Bartl et al. [21] conducted an RCT with 51 patients with prolonged grief disorder (PGD), comparing PG-CBT (a structured 20-session CBT specifically targeting PGD) with a waitlist control. PG-CBT produced significant increases in overall PTGI and all subscales except Spiritual Change, with gains in New Possibilities and Personal Strength maintained at 18-month follow-up [21]. Nijdam et al. [29] compared brief eclectic psychotherapy for PTSD (BEP)—a CBT-related approach incorporating emotional processing, cognitive restructuring, and meaning-making—with EMDR in adult PTSD patients [29]. Both treatments produced significant pre-to-post improvements in all PTGI subscales except Spiritual Change, without significant between-group differences. Importantly, this study lacked a no-treatment control group, limiting causal inference, and the time frame between measurements was not specified.

Three studies applied group cognitive–emotional training—a CBT-based structured program incorporating psychoeducation, normalization of emotional reactions, facilitation of emotional disclosure, self-regulation, and redefinition of personal narrative and life goals through relaxation, mindfulness, cognitive restructuring, and problem-solving techniques—in women with nonmetastatic breast cancer [30] and mothers of children with cancer [31], as well as in a PTG-focused group intervention utilizing CBT strategies [32]. All three studies found significantly higher PTGI scores in intervention groups compared to TAU controls [30,31,32]. Each had small samples and culturally specific populations (Iranian and Portuguese), limiting generalizability.

In contrast, three studies reported no significant relationship between CBT or CBT-related interventions and overall PTG [33,34,35]. Schubert et al. [33] observed no significant changes in total PTGI or subscale scores in 48 PTSD patients assessed at baseline and three months after initiating TF-CBT, though significant improvement was found in the Personal Strengths subscale of the PTGI-SOA (significant other assessment) [33]. The study was limited by the absence of a control group and the short measurement window. Zoellner et al. [34] found no significant increase in total PTG in an RCT of 40 motor vehicle accident survivors receiving CBT versus a waitlist control, although significant increases in New Possibilities and Personal Strength subscales were observed and maintained at follow-up [34]. Ochoa-Arnedo et al. [35] found that neither CBSM nor PPC produced significant overall PTG improvements in 140 female breast cancer survivors; however, PTGI increases in the PPC group predicted better posttraumatic stress outcomes [35]. Critically, none of the three studies with null results employed interventions specifically designed to target PTG.

#### 3.1.2. Internet- and Telephone-Based CBT

Internet-based CBT demonstrated consistent efficacy in fostering PTG across diverse trauma types and populations. All relevant RCTs used the PTGI and compared intervention groups to waitlist controls at matched time points [36,37,38,39]. Treating clinicians were doctoral-level clinical psychologists with specialized training in the respective protocols. Treatment durations ranged from five to ten weeks, with weekly or biweekly sessions incorporating writing assignments, psychoeducation, behavioral activation, breathing techniques, exposure, problem-solving, mindfulness, strength development, and relapse prevention, though none was specifically designed to target PTG.

Knaevelsrud et al. [36] found significant pre-to-post increases in PTGI in adults with post-traumatic stress reactions treated with internet-based CBT versus waitlist control [36]. Wagner et al. [37] reported significant overall PTG increases in patients with complicated grief disorder treated via internet-based CBT compared to controls [37]. Knaevelsrud et al. [38] applied Integrative Testimonial Therapy (ITT)—a 6-week, 11-session internet-based intervention incorporating CBT elements (moderated exposure and cognitive reconstruction) in writing format—to 30 WWII survivors with PTSD symptoms, finding a significant increase in PTG at post-treatment and 3-month follow-up, with a medium effect size [38]. This study used a pre-to-post longitudinal design without a randomized control group, and had a small sample, limiting causal inference. Rachyla et al. [39] demonstrated significant improvement in PTGI in adults with adjustment disorder treated via internet-based CBT plus brief telephone support compared to waitlist controls, with treatment gains stable at 12-month follow-up [39].

An important limitation across these internet-based studies is that participants were recruited primarily via online advertisements and print media, introducing potential selection bias toward individuals with higher educational attainment and media access. Additionally, symptom severity was self-reported in most cases, which may limit precision. Communication between therapists and participants occurred asynchronously via email in all relevant trials.

Chambers et al. [40] compared telephone-delivered cognitive behavioral intervention with a single nurse-led self-management session in 690 cancer patients and caregivers [40]. PTG (measured with the PTGI at baseline, 3, 6, and 12 months) increased in both groups, with no significant between-group difference [40]. The study’s primary limitation is the absence of a no-treatment waitlist control, precluding conclusions about the relative contribution of the active components beyond the passage of time.

### 3.2. PTG Facilitation by CBT in Children, Adolescents, and Young Adults

Evidence for CBT’s beneficial influence on PTG has also been examined in younger populations. Two quasi-experimental studies assessed the effect of Trauma-Focused CBT (TF-CBT) on PTGI scores and emotional management in physically [41,42] and psychologically abused or neglected primary school children [41]. Farnia et al. [41] compared TF-CBT to a control group (TAU) and found significantly increased PTGI scores post-intervention in the TF-CBT group [41]. Salemi et al. [42] compared TF-CBT to a theory-of-mind-based intervention and a control group, finding significantly higher PTG post-intervention in the TF-CBT group relative to both comparators [42]. Limitations common to both studies include small sample sizes, absence of long-term follow-up, culturally limited reference populations (both Iranian), and exclusion of sexual abuse as a trauma condition.

A single RCT examined the effects of Mindfulness-Based Cognitive Therapy (MBCT) on PTG in 64 adolescents and young adults (aged 16–29) with inflammatory bowel disease (IBD) and comorbid depression [43]. Post-traumatic growth was assessed as a secondary outcome at baseline, 8 weeks, and 20 weeks. No significant between-group differences in PTGI were found at either measurement point [43]. This study was substantially limited by a high attrition rate, which reduced statistical power for secondary outcomes, as well as small sample size and lack of randomization stratification by socioeconomic or educational background.

### 3.3. Possible Mechanisms of PTG Facilitation by CBT

Cognitive restructuring [14,15] has been hypothesized as a core mechanism linking CBT to PTG, through the modification of maladaptive trauma-related appraisals and the rebuilding of a more adaptive assumptive worldview. Growth after trauma is considered to result from active cognitive processing, including the challenging and restructuring of core beliefs disrupted by the traumatic experience [8,14,18].

Deliberate rumination—defined as purposeful, reflective processing of traumatic experience, distinct from unconstructive intrusive rumination—has been specifically implicated as a pathway to PTG [8]. CBT may facilitate deliberate rumination through structured therapeutic exercises such as writing assignments (particularly in internet-based formats), cognitive restructuring tasks, and psychoeducation that normalize and scaffold reflective processing.

Meaning-making is another important mechanism [14,16]. Through CBT, individuals may reappraise the meaning of their traumatic experience, explore revised values and life goals, and reframe adversity as an opportunity for personal development. This process may simultaneously reduce posttraumatic stress and promote growth.

Exposure techniques may also foster PTG by promoting approach coping and reducing avoidance, which can itself be a barrier to growth [14,16,17]. Exposure is additionally known to promote cognitive restructuring, through which further growth may be induced.

Self-efficacy has been identified as a predictor of PTG [18,19,20] and may be enhanced through CBT as individuals develop greater mastery over automatic thoughts, emotions, and behaviors via self-regulation and problem-solving skills acquisition. Higher self-efficacy may be associated with more active coping strategies in the face of and following trauma [20].

Finally, reduction in trauma-related symptom severity may serve as a mediator between CBT and PTG. Evidence from Bartl et al. indicates that reduction in prolonged grief symptoms mediated the relationship between PG-CBT and PTG increase [21]. Conversely, higher pre-treatment PTG scores were associated with lower PTSS severity in some studies [33,44,45], suggesting a bidirectional relationship. The co-occurrence of PTG and distress, established in the broader literature [11], suggests that moderate symptom severity may not preclude PTG and may even be a precondition for the cognitive disruption necessary for growth to occur.

The mechanisms presented above are theoretical and have been proposed in the included studies and the broader literature. These mechanisms are not uniformly tested in the included studies; rather, they represent hypotheses that emerge from the evidence reviewed and the PTG theoretical framework. More definitive conclusions regarding mediation await studies with appropriate experimental designs.

## 4. Discussion

This systematic review synthesizes evidence from 19 studies examining the role of CBT and CBT-based interventions in promoting PTG across diverse trauma populations, intervention formats, and delivery modalities. The overall picture that emerges is nuanced: whereas traditional CBT produces inconsistent results with respect to PTG facilitation, CBT-based protocols that incorporate structured cognitive–emotional techniques—particularly those explicitly designed to target PTG—appear more consistently beneficial.

A single RCT including traditional CBT interventions [28] reported significant PTGI increases, while three studies found no significant overall PTG improvement [33,34,35], although some observed gains in specific PTGI subdomains (particularly Personal Strength and New Possibilities). Studies comparing CBT with other active treatments [29,35,42] found no significant superiority of CBT over EMDR, Positive Psychotherapy in Cancer, or nurse-led self-management intervention. This is consistent with meta-analytic conclusions that diverse psychosocial approaches can promote PTG, suggesting that growth may not be uniquely attributable to CBT mechanisms but may be facilitated by a range of meaning-promoting therapeutic processes [12,46].

The certainty of the available evidence must be interpreted with caution. The majority of included studies measured PTG as a secondary outcome, had small sample sizes, and exhibited considerable heterogeneity in intervention type, comparator, population, and outcome measurement timing. Several studies lacked control groups or used waitlist controls that do not account for non-specific therapeutic factors. These methodological limitations, combined with the theoretical complexity of PTG as a construct, suggest that the evidence base remains preliminary. The GRADE-rated certainty of evidence being moderate only for CBT-based PTG-targeted protocols and low overall reflects this status.

An important distinction must be maintained between PTG and symptom reduction. Several studies in this review documented reductions in PTSD symptom severity alongside variable PTG outcomes, reinforcing the conceptual independence of these constructs [7]. PTG represents a qualitative transformation of one’s assumptive world and self-understanding, not merely the absence or reduction in symptoms [5,9]. Interventions that target PTG directly, rather than treating it as an incidental byproduct of distress reduction, appear more likely to produce consistent PTG gains—a finding with direct implications for intervention design.

Another moderating factor of note is resilience. Research suggests that highly resilient individuals may exhibit less PTG after trauma, consistent with the observation that PTG presupposes significant emotional disruption as a catalyst [7,47]. This creates a potential confound in studies that do not control for baseline resilience or prior trauma history. Since PTG achieved through prior adversity may have enhanced resilience, which could in turn attenuate the magnitude of PTG in response to a subsequent trauma, studies should measure PTG both before and after intervention to disentangle treatment effects from dispositional characteristics.

The conceptual complexity of PTG itself merits further discussion. Self-report measures such as the PTGI, used across all included studies, capture perceived growth—the individual’s subjective sense of positive change—which may not always correspond to objectively verified psychological transformation [7,11]. Debate continues in the literature regarding whether PTGI scores reflect genuine schematic change, wishful thinking, coping-related positive reappraisal, or social desirability [11].

Recent developments in CBT for trauma—including third-wave approaches such as Acceptance and Commitment Therapy (ACT), Compassion-Focused Therapy, and integrated trauma-focused protocols—increasingly incorporate meaning-making, values clarification, and psychological flexibility components that map closely onto PTG domains. The potential of these contemporary approaches for PTG facilitation warrants investigation in future research.

Future research regarding the efficacy of CBT in promoting PTG should prioritize extended longitudinal follow-up periods utilizing standardized measurement protocols; this will provide critical clarity on the long-term trajectory of growth post-intervention. Furthermore, given the inherently subjective nature of the PTGI, complementing self-report data with neurobiological markers or objective behavioral indicators could better elucidate the tangible impact of PTG on daily functioning and holistic well-being. Future trials must also systematically account for confounding variables, most notably baseline resilience and pre-treatment symptom severity, to achieve more robust, unconfounded outcomes. Finally, researchers should employ dismantling designs to isolate the specific mechanisms within CBT—such as behavioral activation versus cognitive restructuring—that actively accelerate growth, thereby pinpointing the precise intervention modules required to optimize positive transformation.

These findings yield distinct clinical implications, primarily highlighting the value of prioritizing post-traumatic growth as a formal therapeutic objective alongside traditional symptom mitigation—a dual focus that may substantially improve long-term well-being for trauma survivors [48]. When delivering trauma-focused CBT, practitioners may optimize the likelihood of PTG by deliberately weaving growth-oriented components into standard cognitive behavioral protocols. Such integrations might include targeted psychoeducation on PTG, facilitated deliberate rumination, narrative reconstruction exercises, and the collaborative exploration of personal values and core life goals. Crucially, internet-delivered CBT (iCBT) formats appear promising in this domain. Given their possible efficacy and inherent scalability, these digital modalities may offer a viable pathway to extend PTG-focused trauma interventions to underserved populations who face structural barriers to traditional, in-person clinical care. Ultimately, although further high-quality research is required to definitively establish efficacy, exploring growth-oriented outcomes alongside deficit reduction represents a vital step toward understanding how cognitive behavioral frameworks might best support holistic, long-term trauma recovery.

## 5. Conclusions

This systematic review synthesizes evidence from 20 studies examining the effects of CBT and CBT-based interventions on post-traumatic growth. The available evidence suggests that CBT interventions may be effective in facilitating PTG, with more consistent benefits observed for CBT-based protocols that incorporate structured cognitive–emotional techniques or are specifically designed to target PTG, compared to traditional CBT, which produced mixed results. Internet-based CBT interventions demonstrated particular promise. However, the evidence base is heterogeneous and predominantly preliminary, with most studies measuring PTG as a secondary outcome, limited by small sample sizes, and exhibiting marked variability in intervention type, population, and methodology. The overall certainty of evidence, rated according to the GRADE framework, r. Future research should prioritize multicenter randomized controlled trials with larger samples, uniform protocols, long-term follow-up, and PTG as a primary outcome. Identifying the mechanisms—including deliberate rumination, meaning-making, cognitive restructuring, self-efficacy, and exposure—through which CBT components specifically facilitate PTG, and moderating variables such as trauma type, resilience, and intervention design, will be critical for optimizing clinical outcomes.

## Figures and Tables

**Table 1:** Studies measuring the effect of cognitive behavioral therapy on post-traumatic growth.

Reference	Study Population/Country	Trauma Context	Therapy	Comparator	Outcome/Findings	Study Type
Bartl et al., 2017 [21]	Adults with PGD; Germany	Bereavement	PG-CBT (+ further CBT for comorbidities)	WLC	Significant increase in overall PTGI (F = 6.94, d = 0.60, *p* < 0.05) and all subscales except Spiritual Change. New Possibilities and Personal Strength gains maintained at 18-month follow-up.	RCT
Wagner et al., 2016 [28]	Adults in couples (one with PTSD); USA	Various	CBCT for PTSD	WLC	Significant increase in PTG over time in CBCT group vs. WLC (*p* < 0.05, g = 0.45; moderate effect size). Pre-treatment PTSD symptoms not significantly associated with PTG (*p* > 0.05, g = 0.65).	RCT
Nijdam et al., 2017 [29]	Adult patients with PTSD; Netherlands	Various	BEP	EMDR	Significant increase in total PTGI (t = −5.87, *p* < 0.001, d = 0.656) and all subscales except Spiritual Change, pre- to post-treatment, in both groups; no significant between-group differences (F = 0.06, *p* = 0.809). Baseline PTGI did not predict treatment outcome on IES-R or SI-PTSD.	RCT
Hamidian et al., 2018 [30]	Women with nonmetastatic breast cancer; Iran	Nonmetastatic breast cancer	Group Cognitive–Emotional Training	TAU	Significant difference in mean PTG score between groups 20 weeks after the last session, favoring intervention.	Quasi-Experimental
Shakiba et al., 2019 [31]	Mothers of children with cancer; Iran	Child with cancer	Group Cognitive–Emotional Training	TAU	Significant difference in mean PTG values between groups post-intervention, favoring the intervention (t = 14.90, df = 98, *p* < 0.001).	RCT
Ramos et al., 2017 [32]	Adult women; Portugal	Nonmetastatic breast cancer	PTG-focused group intervention with CBT strategies	TAU	Neither intervention significantly increased PTG (b—0.77, *p* < 0.001) Participants with higher baseline PTG showed lower growth over time (F = 5.86, *p* = 0.017).	RCT
Schubert et al., 2019 [33]	Adults with PTSD; Germany	Various	TF-CBT	Pre-treatment vs. 3 months after start of treatment	No significant changes in total PTGI or subscales at 3 months. Significant increase in PTGI-SOA Personal Strengths subscale. Higher PTGI associated with lower PTSS.	Longitudinal
Zoellner et al., 2010 [34]	Adults with PTSD; Germany	Motor vehicle accident	CBT	WLC	No significant treatment effect on overall PTG (F = 2.13, *p* = 0.15). Significant increases in New Possibilities (*p* = 0.06, d = 0.42) and Personal Strength (*p* = 0.015, d = 0.69) for CBT group, maintained at follow-up.	RCT
Ochoa-Arnedo et al., 2020 [35]	Adult women with breast cancer; Spain	Cancer survivors	CBSM	PPC	Neither intervention significantly improved PTG over time (b = 0.77, *p* = 0.76). Increase in PTGI in PPC group predicted lower post-traumatic stress after treatment.	RCT
Knaevelsrud et al., 2010 [36]	Adults with post-traumatic stress reactions; Germany/international	Various	Internet-based CBT	WLC	Significant increase in PTGI from pre- to post-test in intervention vs. WLC (F = 11.34, *p* < 0.001).	RCT
Wagner et al., 2007 [37]	Adults with complicated grief; Germany	Bereavement	Internet-based CBT	WLC	Significant overall increase in PTG in treatment group vs. WLC. Negative relation between pre-treatment PTG and residual gain in avoidance.	RCT
Knaevelsrud et al., 2014 [38]	Adults with PTSD symptoms (WWII survivors); Germany	WWII-associated trauma	Integrative Testimonial Therapy (ITT)	Pre- to post-treatment comparison	Medium treatment effect size for PTG at post-treatment; significant increase maintained to 3-month follow-up.	Longitudinal
Rachyla et al., 2020 [39]	Adults with adjustment disorder; Spain	Various	Internet-based CBT + brief telephone support	WLC	Significant improvement in PTGI in intervention vs. WLC (F = 23.65, *p* < 0.01). Treatment gains stable at 12-month follow-up (time effect non-significant).	RCT
Chambers et al., 2014 [40]	Cancer patients and caregivers; Australia	Cancer	Cognitive behavioral telephone intervention	Single nurse-led self-management session	PTG increased in both groups over time (baseline, 3, 6, 12 months; d = 0.6–0.64, *p* < 0.05)). No significant between-group difference in PTG.	RCT
Farnia et al., 2017 [41]	Primary school children; Iran	Physical abuse	TF-CBT	Control group (TAU)	Significantly increased PTGI scores post-intervention in TF-CBT vs. control.	Quasi-Experimental
Salemi et al., 2018 [42]	Primary school children; Iran	Physical or psychological abuse/neglect	TF-CBT	Theory of mind intervention + control group	Significantly higher PTG post-intervention in TF-CBT vs. theory of mind group and control.	Quasi-Experimental
Ewais et al., 2021 [43]	Adolescents and young adults with IBD and depression (aged 16–29); Australia	Chronic disease (IBD)	MBCT	TAU	No significant between-group differences in PTGI at 8 or 20 weeks.	RCT
Boettche et al., 2015 [44]	Older adults with PTSD symptoms; Germany	War-related trauma	Internet-based CBT	Delayed treatment group	Higher pre-treatment PTG scores predicted greater reduction in PTSD symptom severity pre- to post-treatment (F = 4.45, *p* = 0.042).	RCT
Hagenaars & van Minnen, 2010 [45]	Adults with PTSD; Netherlands	Various	Prolonged Exposure (PE)	Pre- to post-treatment comparison	Significant pre- to post-treatment increases in PTGI for all subscales except Spiritual Change and Appreciation of Life. Appreciation of Life predicted post-treatment PTSD.	Longitudinal

Abbreviations: BEP = brief eclectic psychotherapy; CBCT = cognitive behavioral conjoint therapy; CBSM = cognitive behavioral stress management; CBT = cognitive behavioral therapy; EMDR = eye movement desensitization and reprocessing; IBD = inflammatory bowel disease; IES-R = Impact of Events Scale–Revised; MBCT = mindfulness-based cognitive therapy; PG-CBT = integrative cognitive behavioral therapy for prolonged grief disorder; PGD = prolonged grief disorder; PPC = Positive Psychotherapy in Cancer; PTG = post-traumatic growth; PTGI = Post-Traumatic Growth Inventory; PTGI-SOA = Post-Traumatic Growth Inventory–Significant Other Assessment; PTSD = post-traumatic stress disorder; SI-PTSD = Structured Interview for PTSD; TAU = treatment as usual; TF-CBT = trauma-focused cognitive behavioral therapy; WLC = waitlist control group.

## Data Availability

No new data was created for this work.

## Supplementary Materials

The supporting information can be downloaded at: https://www.mdpi.com/article/10.3390/healthcare14131857/s1.
