# Supplementary material for: The Relationship Between Cognitive Behavioral Therapy and Post-Traumatic Growth: A Systematic Review
Source: Healthcare (Basel). 2026 Jun 25;14(13):1857. doi: 10.3390/healthcare14131857 (PMC13362499; doi:10.3390/healthcare14131857)
Supplement: Supplementary file 1 [file healthcare-14-01857-s001.zip › healthcare-4257747-supplementary.pdf]

Supplementary Files S1, S2

The Relationship between Cognitive Behavioral Therapy and Post-Traumatic Growth: A Systematic Review

Supplementary File S1

PRISMA 2020 Main Checklist

Topic	No.	Item	Location where item is reported
**TITLE**			
**Title**	1	Identify the report as a systematic review.	Title P.1
**ABSTRACT**			
**Abstract**	2	See the PRISMA 2020 for Abstracts checklist	
**INTRODUCTION**			
**Rationale**	3	Describe the rationale for the review in the context of existing knowledge.	Introduction, P. 1.2
**Objectives**	4	Provide an explicit statement of the objective(s) or question(s) the review addresses.	Introduction, P. 2
**METHODS**			
**Eligibility criteria**	5	Specify the inclusion and exclusion criteria for the review and how studies were grouped for the syntheses.	Materials and Methods, P. 2
**Information sources**	6	Specify all databases, registers, websites, organisations, reference lists and other sources searched or consulted to identify studies. Specify the date when each source was last searched or consulted.	Materials and Methods, P. 2
**Search strategy**	7	Present the full search strategies for all databases, registers and websites, including any filters and limits used.	Materials and Methods, P. 2,3
**Selection process**	8	Specify the methods used to decide whether a study met the inclusion criteria of the review, including how many reviewers screened each record and each report retrieved, whether they worked independently, and if applicable, details of automation tools used in the process.	Materials and Methods, P. 3

Topic	No.	Item	Location where item is reported
**Data collection process**	9	Specify the methods used to collect data from reports, including how many reviewers collected data from each report, whether they worked independently, any processes for obtaining or confirming data from study investigators, and if applicable, details of automation tools used in the process.	Materials and Methods, P. 3
**Data items**	10a	List and define all outcomes for which data were sought. Specify whether all results that were compatible with each outcome domain in each study were sought (e.g. for all measures, time points, analyses), and if not, the methods used to decide which results to collect.	Materials and Methods, P. 3
	10b	List and define all other variables for which data were sought (e.g. participant and intervention characteristics, funding sources). Describe any assumptions made about any missing or unclear information.	Materials and Methods, P. 3
**Study risk of bias assessment**	11	Specify the methods used to assess risk of bias in the included studies, including details of the tool(s) used, how many reviewers assessed each study and whether they worked independently, and if applicable, details of automation tools used in the process.	Materials and Methods, P. 3
**Effect measures**	12	Specify for each outcome the effect measure(s) (e.g. risk ratio, mean difference) used in the synthesis or presentation of results.	Materials and Methods, P. 3
**Synthesis methods**	13a	Describe the processes used to decide which studies were eligible for each synthesis (e.g. tabulating the study intervention characteristics and comparing against the planned groups for each synthesis (item 5)).	Materials and Methods, P. 3
	13b	Describe any methods required to prepare the data for presentation or synthesis, such as handling of missing summary statistics, or data conversions.	Materials and Methods, P. 3
	13c	Describe any methods used to tabulate or visually display results of individual studies and syntheses.	Materials and Methods, P. 3
	13d	Describe any methods used to synthesize results and provide a rationale for the choice(s). If meta-analysis was performed, describe the model(s), method(s) to identify the presence and extent of statistical heterogeneity, and software package(s) used.	Materials and Methods, P. 3
	13e	Describe any methods used to explore possible causes of heterogeneity among study results (e.g. subgroup analysis, meta-regression).	Materials and Methods, P. 3
	13f	Describe any sensitivity analyses conducted to assess robustness of the synthesized results.	Materials and Methods, P. 3
**Reporting bias assessment**	14	Describe any methods used to assess risk of bias due to missing results in a synthesis (arising from reporting biases).	Materials and Methods, P. 3
**Certainty assessment**	15	Describe any methods used to assess certainty (or confidence) in the body of evidence for an outcome.	Materials and Methods, P. 3

Topic	No.	Item	Location where item is reported
**RESULTS**			
**Study selection**	16a	Describe the results of the search and selection process, from the number of records identified in the search to the number of studies included in the review, ideally using a flow diagram.	Results
	16b	Cite studies that might appear to meet the inclusion criteria, but which were excluded, and explain why they were excluded.	Results
**Study characteristics**	17	Cite each included study and present its characteristics.	Results
**Risk of bias in studies**	18	Present assessments of risk of bias for each included study.	Results
**Results of individual studies**	19	For all outcomes, present, for each study: (a) summary statistics for each group (where appropriate) and (b) an effect estimate and its precision (e.g. confidence/credible interval), ideally using structured tables or plots.	Results
**Results of syntheses**	20a	For each synthesis, briefly summarise the characteristics and risk of bias among contributing studies.	Results
	20b	Present results of all statistical syntheses conducted. If meta-analysis was done, present for each the summary estimate and its precision (e.g. confidence/credible interval) and measures of statistical heterogeneity. If comparing groups, describe the direction of the effect.	Results
	20c	Present results of all investigations of possible causes of heterogeneity among study results.	Results
	20d	Present results of all sensitivity analyses conducted to assess the robustness of the synthesized results.	Results
**Reporting biases**	21	Present assessments of risk of bias due to missing results (arising from reporting biases) for each synthesis assessed.	Results
**Certainty of evidence**	22	Present assessments of certainty (or confidence) in the body of evidence for each outcome assessed.	Results
**DISCUSSION**			
**Discussion**	23a	Provide a general interpretation of the results in the context of other evidence.	Discussion, P. 12
	23b	Discuss any limitations of the evidence included in the review.	Discussion, P. 12
	23c	Discuss any limitations of the review processes used.	Discussion, P. 12
	23d	Discuss implications of the results for practice, policy, and future research.	Discussion, P. 12
**OTHER INFORMATION**			

Topic	No.	Item	Location where item is reported
**Registration and protocol**	24a	Provide registration information for the review, including register name and registration number, or state that the review was not registered.	Materials and Methods, P. 2
	24b	Indicate where the review protocol can be accessed, or state that a protocol was not prepared.	Materials and Methods, P. 2
	24c	Describe and explain any amendments to information provided at registration or in the protocol.	P. 13
**Support**	25	Describe sources of financial or non-financial support for the review, and the role of the funders or sponsors in the review.	P. 13
**Competing interests**	26	Declare any competing interests of review authors.	P. 13
**Availability of data, code and other materials**	27	Report which of the following are publicly available and where they can be found: template data collection forms; data extracted from included studies; data used for all analyses; analytic code; any other materials used in the review.	P. 13

PRIMSA Abstract Checklist

Topic	No.	Item	Reported?
**TITLE**			
**Title**	1	Identify the report as a systematic review.	Yes
**BACKGROUND**			
**Objectives**	2	Provide an explicit statement of the main objective(s) or question(s) the review addresses.	Yes
**METHODS**			
**Eligibility criteria**	3	Specify the inclusion and exclusion criteria for the review.	Yes
**Information sources**	4	Specify the information sources (e.g. databases, registers) used to identify studies and the date when each was last searched.	Yes
**Risk of bias**	5	Specify the methods used to assess risk of bias in the included studies.	Yes
**Synthesis of results**	6	Specify the methods used to present and synthesize results.	Yes

Topic	No.	Item	Reported?
**RESULTS**			
**Included studies**	7	Give the total number of included studies and participants and summarise relevant characteristics of studies.	Yes
**Synthesis of results**	8	Present results for main outcomes, preferably indicating the number of included studies and participants for each. If meta-analysis was done, report the summary estimate and confidence/credible interval. If comparing groups, indicate the direction of the effect (i.e. which group is favoured).	Yes
**DISCUSSION**			
**Limitations of evidence**	9	Provide a brief summary of the limitations of the evidence included in the review (e.g. study risk of bias, inconsistency and imprecision).	Yes
**Interpretation**	10	Provide a general interpretation of the results and important implications.	Yes
**OTHER**			
**Funding**	11	Specify the primary source of funding for the review.	No
**Registration**	12	Provide the register name and registration number.	No

From: Page MJ, McKenzie JE, Bossuyt PM, Boutron I, Hoffmann TC, Mulrow CD, et al. The PRISMA 2020 statement: an updated guideline for reporting systematic reviews. MetaArXiv. 2020, September 14. DOI: 10.31222/osf.io/v7gm2. For more information, visit: www.prisma-statement.org

Supplementary File S2.

Table 1. Risk of bias assessment of RCTs, using the Cochrane Risk of Bias 2 (RoB 2) tool

**Domain**	D1: Randomization Process	D2: Deviations from Intended Interventions	D3: Missing Outcome Data	D4: Measurement of the Outcome	D5: Selection of the Reported Result	OVERALL RISK
**Reference**						

Bartl et al. (2017) [21]	**Low Risk:** Randomized controlled trial evaluating integrative CBT.	**Some Concerns:** Waiting list control; participants knew they were not receiving treatment	**Low Risk:** Uses both completer and pooled analysis for long-term stability.	**Some Concerns:** PTG is subjective; waitlist awareness is a standard concern in Domain 4.	**Low Risk:** Clear reporting of trajectories and mediation effects.	**SOME CONCERNS**
Wagner et al. (2016) [28]	**Some Concerns**: Randomization mentioned but concealment details limited in text.	**Some Concerns**: Waitlist design; participants knew they were not in the active group.	**Low Risk**: Data reported for various time points; HLM used to handle nested data.	**Some Concerns**: Outcomes (PTG) are self-reported; waitlist group may respond differently.	**Low Risk**: Primary goal and moderation effects were predefined and reported.	**SOME CONCERNS**
Nijdam et al. (2018) [29]	**Low Risk**: Randomized clinical trial with clear recruitment.	**Low Risk**: ITT analysis used; treatments followed manualized protocols.	**Low Risk**: Dropouts (n=28) did not differ significantly between conditions.	**Low Risk**: Assessors were blind to treatment conditions.	**Low Risk**: Analysis followed a clear plan; results reported for total and subscales.	**LOW**
Shakiba et al. (2019) [31]	**Some Concerns:** Random assignment mentioned, but specific concealment details are limited.	**Some Concerns:** Control group was likely aware of non-intervention status; no blinding possible.	**Low Risk:** Posttest data collected 16 weeks after intervention for both groups.	**Some Concerns:** PTG measured via self-reported Posttraumatic Growth Inventory (PTGI) in an unblinded trial.	**Low Risk:** Results reported for both PTSD and PTG as defined in the study aim.	**SOME CONCERNS**
Ramos et al. (2017) [32]	**Some Concerns:** Large imbalance between groups (n=58 vs. n=147); concealment methods not fully detailed.	**Some Concerns:** No blinding of participants or therapists; group intervention nature.	**Low Risk:** Longitudinal analysis conducted at T1, T2, and T3; missing data handled via Latent Growth Modeling.	**Some Concerns:** PTG is self-reported; knowledge of group assignment could influence subjective ratings.	**Low Risk:** Reported on pre-defined primary and secondary outcomes as per the trial objectives.	**SOME CONCERNS**
Zoellner et al. (2010) [34]	**Some Concerns**: Randomly assigned but minimal details on concealment.	**Some Concerns**: Waitlist design; no blinding of participants possible.	**Low Risk**: Outcome data for the 40 survivors appears complete for the design.	**Some Concerns**: PTG is a self-reported measure; waitlist group awareness is a factor.	**Some Concerns**: Significant results only found in subdomains, increasing risk of selective reporting.	**SOME CONCERNS**
Ochoa-Arnedo et al. (2021) [35]	**Low Risk:** Randomized assignment to active treatment arms.	**Low Risk:** Use of active comparators; ITT analysis employed for deviations.	**Some Concerns:** High attrition rate (~30%), although ITT was used, the reasons for loss could relate to the outcome.	**Some Concerns:** Self-reported PTG; participants in the PTG-specific group may be biased by the intervention's stated goals.	**Low Risk:** Findings reported as intended; detailed results for all measured variables provided.	**SOME CONCERNS**
Knaevelsrud	**Low Risk**: Randomly	**Some Concerns**: Waitlist	**Low Risk**: Attrition (16%	**Some Concerns**: Outcomes	**Low Risk**: Comprehensive	**SOME**

et al. (2010) [36]	assigned ; no significant baseline differences found.	design; participants knew they were not receiving treatment.	in Tx, 2% in WLC) is reported and typical for internet-based trials.	are self-reported; awareness of treatment status can affect responses.	reporting of pre-defined outcomes including PTG, openness, and optimism.	**CONCERNS**
Wagner et al. (2007) [36]	**Low Risk**: Randomly assigned; part of a larger established RCT protocol.	**Some Concerns**: Waitlist design; participants aware of their group assignment.	**Low Risk**: Data appears complete for the randomized sample analyzed.	**Some Concerns**: Self-reported PTGI; no blinding of participants possible in waitlist design.	**Low Risk**: Follows the protocol of the larger trial; reports both significant and non-significant (optimism) results.	**SOME CONCERNS**
Rachyla et al. (2020) [39]	**Low Risk:** Participants were randomly allocated to two equal-sized groups (n=34 each).	**Some Concerns:** Waiting list control design; participants knew they were not receiving the active treatment.	**Low Risk:** 76.5% completion rate; gains maintained at 3-, 6-, and 12-month follow-ups.	**Some Concerns:** Self-reported PTG; participants' awareness of treatment group is a factor.	**Low Risk:** Both primary (depression/anxiety) and secondary (PTG, QoL) outcomes are reported.	**SOME CONCERNS**
Chambers et al. (2014) [40]	**Low Risk:** Computer-generated sequence; allocation concealed from recruiters.	**Low Risk:** Both groups received active interventions; protocol deviations were minimal.	**Low Risk:** Attrition was documented and did not differ significantly between arms; appropriate statistical handling.	**Low Risk:** Self-report measures were used, but the comparison between two active interventions reduces bias.	**Low Risk:** Analysis followed the trial registration and reported all primary/secondary outcomes.	**LOW**
Ewais et al. (2021) [43]	**Low Risk:** Randomized allocation to MBCT or TAU.	**Some Concerns:** MBCT vs. TAU; no blinding of participants possible.	**Low Risk:** ITT analysis used to account for the 30% attrition rate.	**Some Concerns:** Self-reported PTG in an unblinded pilot trial.	**Low Risk:** Reports on both primary (depression) and secondary (PTG) outcomes.	**SOME CONCERNS**
Böttche et al. (2015) [44]	**Some Concerns**: Randomized with immediate/delayed groups; concealment details limited.	**Some Concerns**: Delayed treatment design; participants were aware of their group status.	**Low Risk**: Follow-up assessments were conducted; results reported for the completed sample.	**Some Concerns**: PTG is a self-reported measure; knowledge of group assignment could influence responses.	**Low Risk**: Reports on pre-treatment predictors and post-treatment outcomes as planned.	**SOME CONCERNS**

Table 2. Risk of Bias (ROBINS-I) – Quasi-Experimental Studies

Study	Confounding	Selection of Participants	Classification of Interventions	Deviations from Intended Interventions	Missing Data	Measurement of Outcomes	Selection of Reported Results	Overall Risk of Bias
Hamidian et al., 2018 [30]	Moderate	Moderate	Low	Moderate	Low	Low	Moderate	Moderate
Farina et al., 2017 [41]	Moderate	Moderate	Low	Moderate	Low	Moderate	Moderate	Moderate
Salemi, S al., 2018 [42]	Serious	Moderate	Low	Moderate	Low	Moderate	Moderate	Serious

Table 3. Risk of Bias Assessment — Newcastle-Ottawa Scale (NOS)

Study	Selection				Comparability		Outcome				Total Stars
	S1 Representativeness	S2 Non-exposed Selection	S3 Exposure Ascertainment	S4 Outcome-free at Start	C1 Comparability (Design/Analysis)	C2 Comparability (Confounders)	O1 Outcome Assessment	O2 Follow-up Duration	O3 Follow-up Adequacy	O4 Blinding of Assessors	/ 9
**Schubert et al. (in press) [33]**	★	☆	★	★	★	☆	★	★	★	☆	**6 / 9**
**Knaevelsrud et al. (2014) [38]**	★	☆	★	★	☆	☆	★	★	★	☆	**6 / 9**
**Hagenaars & van Minnen (2010) [45]**	★	☆	★	★	★	☆	★	★	★	☆	**7 / 9**
**Criterion Description**	Repr. of treated sample	Appropriate comparison / control	Standardised exposure ascertainment	Outcome not present at baseline	Controlled for main confounders	Additional confounders controlled	Validated outcome measures	Follow-up sufficient	Adequate follow-up rate	Independent / blinded assessment	**Max: 9**

Legend & Scoring Rationale

★ = criterion met (1 star awarded) ☆ = criterion not met (0 stars) C1 = up to 2 stars for comparability

S1 – Representativeness: ★ if sample derived from a defined clinical population with diagnostic confirmation. S2 – Non-exposed selection: ★ if a comparator/control group present; neither study had a control group (☆). S3 – Exposure ascertainment: ★ if treatment protocol was standardised and fidelity monitored. S4 – Outcome-free at start: ★ if diagnosis confirmed before treatment (PTG not yet expected from therapy).

C1 – Comparability (design): ★ if study controlled statistically for key covariate (e.g., baseline PTSS). C2 – Additional confounders: ★ if further confounders addressed (e.g., randomisation, comorbidity adjustment); not met in either study.

O1 – Outcome assessment: ★ if validated, psychometrically sound instruments used. O2 – Follow-up duration: ★ if sufficient for meaningful symptom change. O3 – Follow-up adequacy: ★ if ≥70% retention or adequate dropout analysis. O4 – Blinding: ★ if outcome assessors were independent/blind to treatment; neither study met this (☆).

Overall interpretation: ≥7 stars = low risk; 5–6 = moderate risk; ≤4 = high risk of bias.

Table 4. GRADE assessment of included studies

Author(s): Dimitrios Kasimis, Paschalia Mitskidou, Athanasios Tselebis, Ioannis Ilias, Argyro Pachi
Question: Effectiveness of CBT and CBT-related interventions in promoting PTG
Setting: Community; general population

Bibliography: see References section.

Certainty assessment							№ of patients		Effect		Certainty	Importance
№ of studies	Study design	Risk of bias	Inconsistency	Indirectness	Imprecision	Other considerations	CBT or CBT-related treatment protocols	no CBT intervention (WLC, TAU, different treatment modality)	Relative (95% CI)	Absolute (95% CI)		
**PTGI score increase for Traditional CBT (assessed with: PTGI)**												
3	randomised trials	serious[a]	serious[b]	not serious	not serious	none	159	149	-	see comment	⊕⊕◯◯ Low[a,b]	
**PTGI score increase for internet or telephone based CBT (assessed with: PTGI)**												
6	randomised trials	serious[a]	not serious	not serious	not serious[c]	none	520	516	-	see comment	⊕⊕⊕◯ Moderate[a,c]	
**PTGI score increase for PTG-focused CBT-related protocols (assessed with: PTGI)**												
4	randomised trials	serious[a]	not serious	not serious	not serious	none	175	269	-	see comment	⊕⊕⊕◯ Moderate[a]	
**PTGI score increase in non-randomized studies (assessed with: PTGI)**												
6	non-randomised studies	serious[d]	serious[e]	not serious	not serious	all plausible residual confounding would suggest spurious effect, while no effect was observed	209	68	-	see comment	⊕◯◯◯ Very low[d,e]	

CI: confidence interval

Explanations

a. Some concerns in reporting of allocation concealment, some concerns in deviations from intended interventions due to WLC, some concerns in measurement of outcome due to self-report PTGI scale.
b. Heterogeneity across intervention methods, study designs and measurement of PTG as either a primary or secondary outcome, results in downgrading consistency by 1 level.
c. Self-reporting of symptom severity via online forms might suggest lower precision, but with no direct impact on PTG measurement; precision level was not reduced.
d. Absence of randomiz ation and limited control for confounders in quasi-experimental studies as well as lack of control group condition in cohort studies imply serious risk of bias.
e. Heterogeneity in treatment protocols, study designs and primary outcomes measured between studies result in downgrading consistency by 1 level.
